# Long-term prospective longitudinal evaluation of emotional distress and quality of life in cervical cancer patients who remained disease-free 2-years from diagnosis

**DOI:** 10.1186/1471-2407-13-127

**Published:** 2013-03-18

**Authors:** Giovanna Mantegna, Marco Petrillo, Gilda Fuoco, Laura Venditti, Serena Terzano, Luigi Pedone Anchora, Giovanni Scambia, Gabriella Ferrandina

**Affiliations:** 1 Psycho-Oncology Service, Catholic University, Campobasso, Italy; 2 Department of Obstetrics and Gynecology, Gynecologic Oncology Unit, Catholic University Rome, Rome, Italy; 3 Gynecologic Oncology Unit, Department of Oncology, Catholic University, Campobasso, Italy

**Keywords:** Quality of life, Emotional distress, Cervical cancer, Prospective study, Long-term evaluation

## Abstract

**Background:**

A long-term prospective assessment of QoL in cervical cancer patients is still lacking. Here, we provide the first 2-years prospective, longitudinal study evaluating emotional distress and QoL in early stage (ECC) and locally advanced (LACC) cervical cancer patients who remained disease-free 2-years from diagnosis.

**Methods:**

The questionnaires: Hospital Anxiety and Depression Scale (HADS), Global Health Status items of EORTC QLQ-C30 (GHS), and EORTC QLQ-CX24 (CX24) have been administered by a dedicated team of psycho-oncologists, administered at baseline, and after 3, 6, 12 and 24 months from surgery The Generalized Linear Model for repeated measure was used to analyze modifications of QoL measures over time.

**Results:**

In both groups, an early reduction of the percentage of patients with anxiety levels ≥11 was observed at the 3-month evaluation (ECC: 25.7% at baseline Vs 14.7% after 3 months, p value=0.001; LACC: 22.2% at baseline Vs 15.4% after 3 months, p value=0.001). Despite this favorable trend, after 2 years from diagnosis, 11.9% of ECC and 15.6% of LACC patients still showed an anxiety score ≥11. No significant changes over time were observed in term of Depression levels. Focusing on QoL issues, mean GHS and Sexual Activity scores showed an improvement over time in both groups compared to baseline (GHS: 5.7% difference for ECC, p value=0.001, and 11.0% in LACC, p value=0.001; SXA: 13.9% difference for ECC, p value=0.001; and 6.1% in LACC, p value=0.008). On the other hand, Body Image mean scores were significantly impaired by chemoradiation administration in LACC patients, without long-term recovery (7.5% difference, p value=0.001). Finally, in both groups, lymphedema (LY) and menopausal symptoms (MS) showed an early worsening which persisted 2-year after surgery (LY: 19.5% difference for ECC, p value=0.014, and 27.3% in LACC, p value=0.001; MS: 14.4% difference for ECC, p value=0.004, and 16.0% in LACC, p value=0.002).

**Conclusions:**

Despite a significant improvement over time, elevated anxiety levels are still detectable at 24 months after surgery in approximately 10% of cervical cancer patients. Much more attention should be focused on surgical/medical approaches able to minimize the negative impact of lymphedema and menopausal symptoms on QoL.

## Background

Over the last decade, increasing attention has been focused on the issues of emotional distress and quality of life (QoL) in women with gynecological cancer [1,2]. This renewed attention comes from the growing awareness that cancer diagnosis and the consequences of multimodal treatments deeply affect woman’s self-identity, impairing her social/intimate relationships, as well as her overall self-perception as mother and wife [3-5]. These considerations are supported by prevalence studies, which estimated that a new diagnosis of gynecological cancer is associated with moderate/severe anxiety or depression in about 50% of women [6,7].

In particular, among gynecological cancer patients, those affected by cervical cancer have been reported to show the worst scores in terms of emotional distress and QoL [4,5,8], given their younger age, and the need to undergo integrated therapies and aggressive surgical procedures [9,10]. Moreover, several experiences suggest that anxiety, depression and QoL deterioration persist for months to years since diagnosis, but the variables influencing the time interval to spontaneous recovery are far from being clarified [11-15].

The available literature on this specific field shows a large variability, and it can be justified by the retrospective nature of the studies, which are often cross-sectional, performed at different time intervals from treatment and lacking of baseline evaluations. For these reasons, we have performed a prospective and longitudinal collection of data on QoL levels in early (ECC) and locally advanced (LACC) cervical cancer patients treated with radical surgery (RS) or chemoradiation (CT/RT) followed by RS, respectively [16].

Here, we provide an updated analysis of previously published data, describing the longitudinal modifications of anxiety/depression and QoL scores, in a large cohort of cervical cancer patients who remained disease-free 2 years from diagnosis. We identify in this study also the clinico-pathological and socio-demographic features influencing emotional distress and QoL levels.

## Methods

### Study design and recruitment

We conducted in our Institution, a prospective, longitudinal study, collecting specific measures of emotional distress and QoL in a large cohort of cervical cancer patients. Eligibility criteria include: histological diagnosis of cervical carcinoma, age≥18 years, ability to read and understand Italian language, and absence of any evident cognitive impairment. The study was approved by the Institutional Review Board, and by the Ethical Committee of the Catholic University of the Sacred Heart. All ECC patients (FIGO Stage IB-IIA <4 cm tumor size) were treated with radical hysterectomy plus pelvic lymphadenectomy, while LACC patients (FIGO stage IB-IIA >4 cm tumor size, IIB-IVA) received concomitant chemoradiation (CT/RT) followed by RS in case of response, as previously reported [17-19].

### Measures

Symptoms of anxiety and depression were evaluated with the Italian validated version of the Hospital Anxiety and Depression Scale (HADS including 14 items) questionnaire [20,21]. Responses were provided on verbal scales coded 0–3, and were grouped as follows: 0–7=normal, 8–10=borderline, 11–21=abnormal, according to the original version of HADS validated by Zigmond et al. [21].

Patients QoL was assessed administering the Global Health Status scale of EORTC QLQ-C30 (version 3.0) (GHS including 2 items) and the EORTC QLQ-CX24 (CX24 including 22 items) questionnaires [22,23]. Scores were obtained after a linear transformation of data according to the procedures validated by the EORTC QoL Group [22,23]. Higher scores on the GHS and Sexual activity subscales indicate a higher level of functioning and a better QoL, whereas, for the remaining subscales (i.e. HADS, Symptom experience, Body image, Lymphedema, Menopausal symptoms), higher levels correspond to worse or more symptoms.

EORTC published guidelines were applied for the interpretation of clinical relevant changes of GHS scores [24]. Furthermore, as previously reported, a difference larger than 5% of mean score values compared to baseline was considered as indicating a difference of clinical interest for the EORTC QLQ-CX24 and HADS subscales [25,26].

### Study procedures

Written informed consent to the study procedures was obtained from all eligible patients. Baseline questionnaires were administered within a week from communication of diagnosis and before any counseling about treatment. Patients had also to complete the questionnaires 3, 6, 12 and 24 months after surgery. For LACC patients an additional time point for QoL assessment was planned after 4–5 weeks from completion of chemoradiation. QoL evaluations were discontinued in patients experiencing recurrence/progression of disease. All questionnaires were administered by psycho-oncologists from our hospital service.

### Statistical analysis

Only patients who completed all questionnaires, at all time points, were considered eligible and included in the final analysis. All analyses were performed separately in ECC and LACC groups in order to reduce sample heterogeneity. ANOVA was used to analyze the baseline differences of QoL scores according to group of disease. Categorical variable were examined using the Fisher’s exact test. The Generalized Linear Model (GLM) for repeated measure ANOVA test was used to analyze modifications of QoL measures over time: a p value< 0.05 was taken as statistically significant. The Between Subject test was used to investigate the association between factors and the changes over time in term of patients’ anxiety scores. The SPSS Software 18 (SPSS Inc., Chicago, IL) was used to carry on statistical analysis.

## Results

### Compliance

**Figure 1:**
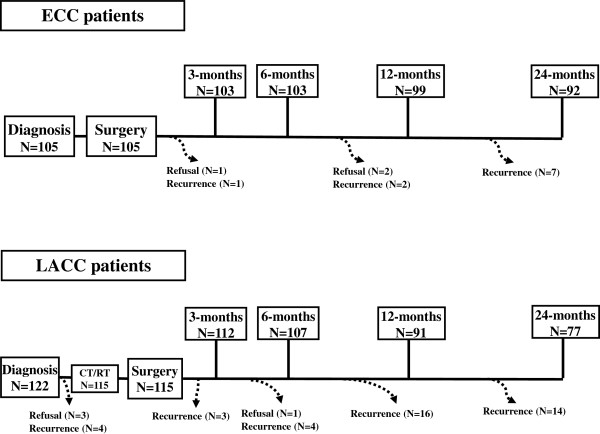
**Time line of questionnaire administration**, **and flow chart of our patient population.**

Between February 2007 and July 2010, 227 patients were enrolled in the study (Figure 1). During the study timeframe, we observed 10 (9.5%), and 41 (33.6%) recurrences, in ECC and LACC patients, respectively; 3 (2.8%) ECC and 4 LACC (3.3%) women refused to complete all study evaluations. Therefore, 92 ECC and 77 LACC patients were considered eligible for final analysis (overall study compliance=96.1%).

### Characteristics and socio-demographics features

As shown in Table 1, at diagnosis, LACC patients resulted older and more frequently postmenopausal than ECC patients. We did not observe differences, in terms of co-morbidities and adjuvant therapies, between the two groups. The vast majority of patients was married (63.8%), lived with someone (87.6%), had children (80.2%), and a higher education level (84.1%); on the other hand, 51.7% of women were unemployed/retired. No statistically significant differences were detected in the distribution of clinico-pathological and socio demographics features between eligible patients and women excluded from the final analysis due to refusal or recurrence/progressive disease.

**Table 1:** **Characteristics and socio**-**demographic features according to stage of disease**

**Characteristics**	**Total No. (%)**	**Early stage cervical cancer No. (%)**	**Locally advanced cervical cancer No. (%)**	***p*value**^**a**^
**Whole series**	227	105 (46.3)	122 (53.7)	-
**Age**, **years**				
Median (range)	50.0 (27.3-82.0)	46.1 (27.3-82.0)	52.5 (29.1-81.2)	**0**.**001**^**b**^
**Menopause** (at diagnosis)				
No	124 (54.6)	68 (64.8)	56 (45.9)	
Yes	103 (45.4)	37 (35.2)	66 (54.1)	**0**.**006**
**Co**-**morbid conditions**				
No	173 (76.2)	84 (80.0)	89 (72.9)	
Yes	54 (23.8)	22 (20.0)	32 (27.1)	0.234
**Adjuvant Therapy**				
None	162 (71.3)	77 (73.3)	85 (69.6)	
Yes	65 (28.7)	28 (26.7)	37 (30.4)	0.312
**Use of antidepressant drugs**				
No	193 (85.1)	90 (85.7)	103 (84.4)	
Yes	34 (14.9)	15 (14.3)	19 (15.6)	0.853
**Marital Status**				
Married	145 (63.8)	70 (66.7)	75 (61.4)	
Not married/widowed/divorced	82 (36.2)	35 (33.3)	47 (38.6)	0.483
**Living status**				
Alone	28 (12.3)	10 (9.5)	18 (14.7)	
Not alone	199 (87.7)	95 (90.5)	104 (85.3)	0.312
**Children**				
No	45 (19.8)	19 (18.1)	26 (21.3)	
Yes	182 (80.2)	86 (81.9)	96 (78.7)	0.614
**Education level**				
Primary school	36 (15.8)	12 (11.4)	24 (19.7)	
High school/graduation)	191 (84.2)	93 (88.6)	98 (80.3)	0.102
**Employment status**				
Employed	111 (48.9)	49 (46.7)	62 (50.8)	
Unemployed/retired	116 (51.1)	56 (53.3)	60 (49.2)	0.623

^a^ calculated by Fisher’s exact test for proportion.

^b^ calculated by Wilcoxon rank sum non parametric test.

### Anxiety and depression

**Figure 2:**
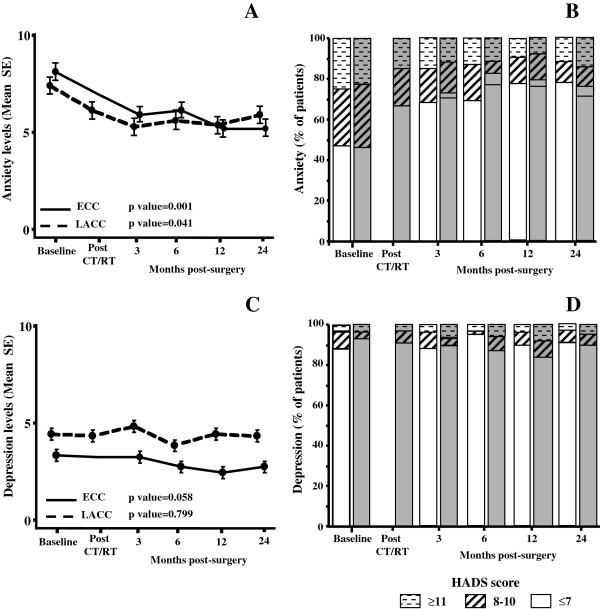
**Plots of HADS-anxiety (A) and depression (C) mean scores at each assessment (Error bars indicate standard deviations).** Longitudinal changes over time in the distribution of patients with normal (HADS scores≤7), borderline (HADS scores 8–10), and severe (HADS scores ≥11) levels of anxiety (**B**) and depression (**D**) according to the extent of disease; ECC patients: white column, LACC patients: gray columns.

Longitudinal changes of anxiety and depression scores are reported in Figure 2. At baseline, mean anxiety levels did not significantly differ between ECC and LACC patients (p value=0.443). Both groups experienced a statistically significant early (3 months after surgery) improvement of anxiety scores, which persisted until the 24-month evaluation (Figure 2A).

As shown in Figure 2B, at baseline, an anxiety score ≥11 was documented in 25.7% and 22.2% of ECC and LACC patients, respectively. A statistically significant reduction of the percentage of patients with severe anxiety levels was observed at the 3-month evaluation in both groups (ECC=14.7%, p value=0.001; LACC=15.4%, p value=0.001); but, after 2 years from diagnosis, 11.9% and 15.6% of ECC and LACC patients still showed an anxiety score ≥11.

As far as depression is concerned, low and comparable mean scores were detected at baseline in both ECC and LACC patients (p value=0.669), with only 3% of patients showing HADS-depression levels ≥11. We did not find any change of mean depression scores or distribution of cases with pathological depression scores over time (Figure 2C, D).

Univariate analysis showed that ECC patients, who were not in menopause at diagnosis, had worse anxiety scores, although this association went lost in multivariate analysis. On the other hand, in LACC patients, multivariate analysis showed that only *living status not alone* was significantly associated with higher anxiety scores (Table 2).

**Table 2:** **Univariate and multivariate analysis of the association between socio**-**demographic features and changes over time of anxiety scores in ECC and LACC patients**

**Independent variable**	**ECC**			**LACC**		
	**Association with higher anxiety scores**	**Univariate (*p value*)**	**Multivariate**^**a **^**(*p value*)**	**Association with higher anxiety scores**	**Univariate (*p value*)**	**Multivariate (*p value*)**
**Age**						
≤45 years	No difference	0.971	/	No difference	0.371	/
>45 years						
**Menopausal status**						
No	**Yes** (**direct**)	**0**.**056**	0.242	No difference	0.254	/
Yes	**No**					
**Co**-**morbidities**						
No	No difference	0.701	/	No difference	0.820	/
Yes						
**Marital status**						
Married	No difference	0.105	0.270	No difference	0.300	/
Not married						
**Living status**						
Alone	No difference	0.114	0.733	**No**	**0**.**023**	**0**.**002**
Not alone				**Yes** (**direct**)		
**Children**						
No	No difference	0.332	/	No difference	0.215	0.635
Yes						
**Educational level**						
Primary school	No difference	0.114	0.225	**Yes** (**direct**)	**0**.**040**	0.773
High/graduation				**No**		
**Employment status**						
Employed	No difference	0.763	/	No difference	0.623	/
Unemployed/retired						

^*a*^ p values calculated by the Between Subject test including in multivariate analysis all variables showing a *p value*≤*0*.*20* in univariate analysis (Generalized linear model).

As far as depression scores is concerned, there was no association between patients’ characteristics and depression levels, both in univariate and multivariate analysis (data not shown).

### QoL subscales

**Figure 3:**
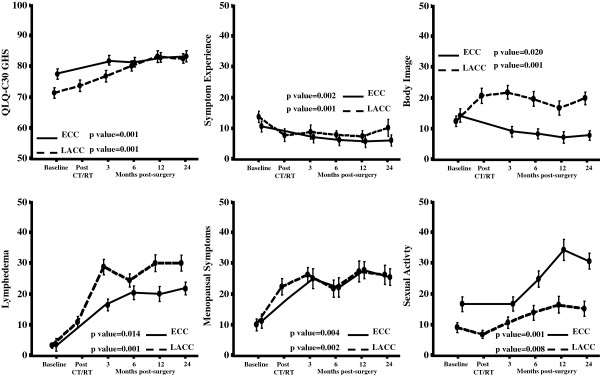
Plots of the QoL scales/items showing statistically significant variations over time (Error bars indicate standard deviations).

As shown in Figure 3, in ECC patients, GHS scores showed a slight clinically significant improvement over time (5.7% difference in mean score compared to baseline); similarly a clinically and statistically significant improvement of GHS scores (11.0% difference in mean score compared to baseline) was documented in LACC patients at the 24-month compared to baseline evaluation (p value=0.001).

Regarding symptom experience (SE), the difference of mean scores was never clinically significant (less than 5%).

On the other hand, differently from ECC patients, who experienced a little but progressive improvement of body image (BI) scores over time, LACC patients showed a relevant deterioration of BI levels, which arose shortly after CT/RT administration and did not show a return to basal levels even after 2 years since diagnosis.

As far as lymphedema (LY) is concerned, we observed a dramatic and persistent worsening of mean scores over time both in ECC (difference of 19.5 in mean values compared to baseline at 24 months from surgery, p value=0.014), and LACC cases (difference of 27.3 in mean values compared to baseline at 24 months from surgery, p value=0.001).

Similarly, we observed in both groups a significant deterioration of scores of menopausal symptoms (MS), without a recovery after 24 months from surgery.

Finally, scores relative to sexual activity showed a marked improvement over time with a difference of mean score values compared to baseline of 13.9% for ECC (p value=0.001), and 6.1% for LACC patients (p value=0.008).

In ECC patients, the only association retained in multivariate analysis was between premenopausal status at diagnosis and worse menopausal symptoms (p value=0.001). In LACC patients, the only statistically significant association, confirmed in multivariate analysis, was detected between *having children* and poor GHS score values (p value=0.053) (data not shown).

## Discussion

We reported for the first time a long-term, prospective, longitudinal evaluation of emotional distress and QoL in a large series of women with ECC and LACC. During the whole study period, around one thousand of questionnaires have been successfully administered with a relevant patients’ compliance to the study (96.1%). This low “refusal rate” could reflect the appreciation of patients for the efforts made to bring out their unmet needs; furthermore, administration of questionnaires could have been seen by the patients as a precious opportunity for processing their own disease experiences.

Looking at emotional issues, we reported baseline severe HADS-anxiety scores in approximately 20-25% of patients, confirming previously published data [6,7,27-29]. Moreover, there were no differences in baseline anxiety scores between ECC and LACC patients; thus suggesting that, since baseline evaluation was administered before any counseling about treatment, the development of severe anxiety could be mainly related to the communication of cancer diagnosis, rather than to concerns regarding the extent of disease and the treatment modalities.

We documented a significant improvement of anxiety levels in both groups at the 3-month evaluation, confirming that the first 100 days after diagnosis play a crucial role in the process of emotional recovery [11,26]. Therefore, it is not surprising that interventions on psychological symptoms have been primarily investigated in the early phase after cancer diagnosis [28,30]. However, it has to considered that in our study, anxiety levels reached a plateau over 2 years, and severe anxiety scores were still detectable in 10-15% of patients. Similar results were reported by Wenzel et al., who documented persistent psychological sequelae in a large series of long-term survivors, with around 50% of respondents expressing the need to be involved in counseling programmes, in order to discuss psychosocial issues raised by having had cervical cancer [15].

In this context, it emerges an urgent need to identify those women at risk for persistent emotional distress. In particular, we observed that ECC patients who were not menopausal at time of diagnosis showed worse anxiety scores. Furthermore, in LACC cases, *low educational level* and *living status not alone* were associated with higher anxiety scores. As explanation to the observed association between the *living not alone* and higher anxiety levels, we can infer that, those cancer patients, who lives in a family context, could have to cope with fear of being less able to sustain major responsibilities from children management and partner relationship. Therefore, it has to be emphasized that prevention or reduction of anxiety levels would require social and family interventions.

As far as QoL issues are concerned, a gradual, progressive improvement of GHS was observed within the first year from diagnosis, with stable levels at 24-month evaluation, suggesting that cervical cancer patients require a relatively brief time interval to come and accommodate with their illness, adjusting personal internal standard for QoL measures.

As reported in our preliminary experience [16], lymphedema and menopausal symptoms emerged as the most important, long term parameters affecting patients QoL. The administration of radical pelvic surgery combined with radiotherapy plays a crucial role in the development of these symptoms, as also sustained by the close temporal association between the emergence of poor BI scores and the employment of chemoradiation in the group of LACC patients. Furthermore, the extent and persistence of QoL deterioration in women with lymphedema and menopausal symptoms emphasize the need to triage cervical patients to dedicated multidisciplinary teams, during the early phase after completion of primary treatment.

Radical pelvic surgery and chemoradiation are obviously associated with a significant impairment of sexual functioning in cervical cancer patients [4,12,13]. However, we observed a clinically relevant improvement of sexual activity scores over time, regardless of disease extent. Reasons supporting our findings could be the introduction of more tailored procedures in surgical management [31], and also the lack of brachytherapy which resulted in a lower dose of total radiation to genital tract, compared to conventional approaches. On the other hand, it has to be emphasized that, despite a similar trend of recovery, women with LACC showed significantly lower levels of sexual activity compared to ECC patients (Figure 3).

## Conclusions

In conclusion, our experience revealed a gradual improvement of emotional distress and QoL issues during the first 2 years after diagnosis of cervical cancer, with the exception of LY and MS symptoms. It is conceivable that long-term improvement of emotional issues could be due mainly due to a higher patient confidence in the successful management of disease. However, it has to be acknowledged that also psychological mechanisms of coping and adaptation may contribute to time interval and extent of resumption from acute emotional disruption. The relative lack of association in multivariate analysis between independent variables and emotional distress, underlines that each patient exerts over time her own personal capabilities to rescue over time form anxiety and QoL deterioration.

A prospective study (IRIS-2, I.R.I.S., http://www.iris-og-com) is going to be launched in our Institution to assess the impact of early and long term psychological interventions on emotional and QoL distress, as well as on immune functions in cervical cancer patients.

## Competing interests

The authors declare that they have no competing interests.

## Authors’ contributions

GM: contributed to the conception and design of the study, analysis and interpretation of the results, drafting of the final manuscript. MP: contributed to the analysis and interpretation of the results, drafting of the final manuscript, and preparation of table and figures. GFu: contributed to the data collection and table and figures preparation. LV: contributed to the data collection and table and figures preparation. ST: contributed to the data collection and table and figures preparation. LPA: contributed to the analysis and interpretation of the results, drafting of the final manuscript. GS: contributed to the conception, design of the study, interpretation of the results, drafting of the final manuscript. GFe: contributed to the conception, design of the study, interpretation of the results, drafting of the final manuscript. All authors read and approved the final manuscript.

## Pre-publication history

The pre-publication history for this paper can be accessed here:

http://www.biomedcentral.com/1471-2407/13/127/prepub

## References

[B1] Vistad I Fossa SD Dahl AA A critical review of patient-rated quality of life studies of long-term survivors of cervical cancer Gynecol Oncol 2006 102 563 572 10.1016/j.ygyno.2006.03.050 16716379

[B2] Chase DM Watanabe T Monk BJ Assessment and significance of quality of life in women with gynecologic cancer Future Oncol 2010 6 1279 1287 10.2217/fon.10.96 20799874

[B3] Juraskova I Butow P Robertson R Sharpe L McLeod C Hacker N Post-treatment sexual adjustment following cervical and endometrial cancer: a qualitative insight Psycho-Oncol 2003 12 267 279 10.1002/pon.639 12673810

[B4] Park SY Bae DS Nam JH Park CT Cho CH Lee JM Lee MK Kim SH Park SM Yun YH Quality of life and sexual problems in cervical cancer survivors Cancer 2007 110 2716 2725 10.1002/cncr.23094 17960806

[B5] Korfage IJ Essink-Bot ML Mols F van de Poll-Franse L Kruitwagen R van Ballegooijen M Health-related quality of life in cervical cancer survivors: a population-based survey Int J Radiat Oncol Biol Phys 2009 73 1501 1509 10.1016/j.ijrobp.2008.06.1905 18823716

[B6] Evans DW McCartney CF Nemeroff CB Raft D Quade D Golden RN Haggerty JJ Jr Holmes V Simon JS Droba M Depression in women treated for gynaecological cancer: clinical and neuroendocrine assessment Am J Psychiatry 1986 143 447 452 3082223 10.1176/ajp.143.4.447

[B7] Cain EN Kohorn EI Quinlan DM Schwartz PE Latimer K Rogers L Psychosocial reactions to the diagnosis of gynecologic cancer Obstet Gynecol 1983 62 635 641 6621954

[B8] Distefano M Riccardi S Capelli G Costantini B Petrillo M Ricci C Scambia G Ferrandina G Quality of life and psychological distress in locally advanced cervical cancer patients administered pre-operative chemoradiotherapy Gynecol Oncol 2008 111 144 150 18692225 10.1016/j.ygyno.2008.06.034

[B9] Ferrandina G Margariti PA Smaniotto D Petrillo M Salerno MG Fagotti A Macchia G Morganti AG Cellini N Scambia G Long-term analysis of clinical outcome and complications in locally advanced cervical cancer patients administered concomitant chemoradiation followed by radical surgery Gynecol Oncol 2010 119 404 410 10.1016/j.ygyno.2010.08.004 20817228

[B10] Chemoradiotherapy for Cervical Cancer Meta-analysis Collaboration (CCCMAC) Reducing uncertainties about the effects of chemoradiotherapy for cervical cancer: individual patient data meta-analysis Cochrane Database Syst Rev 2010 1 CD008285 20091664 10.1002/14651858.CD008285 PMC7105912

[B11] Weisman AD Worden JW The existential plight in cancer: significance of the first 100 days Int J Psychiatry Med 1976 7 1 15 10.2190/UQ2G-UGV1-3PPC-6387 1052080

[B12] Frumovitz M Sun CC Schover LR Munsell MF Jhingran A Wharton JT Eifel P Bevers TB Levenback CF Gershenson DM Bodurka DC Quality of life and sexual functioning in cervical cancer survivors J Clin Oncol 2005 23 7428 7436 10.1200/JCO.2004.00.3996 16234510

[B13] Greimel ER Winter R Kapp KS Haas J Quality of life and sexual functioning after cervical cancer treatment: a long-term follow-up study Psycho-Oncol 2009 18 476 482 10.1002/pon.1426 18702067

[B14] Kobayashi M Ohno T Noguchi W Matsuda A Matsushima E Kato S Tsujii H Psychological distress and quality of life in cervical cancer survivors after radiotherapy: do treatment modalities, disease stage, and self-esteem influence outcomes? Int J Gynecol Cancer 2009 19 1264 1268 10.1111/IGC.0b013e3181a3e124 19820389

[B15] Wenzel L DeAlba I Habbal R Kluhsman BC Fairclough D Krebs LU Anton-Culver H Berkowitz R Aziz N Quality of life in long-term cervical cancer survivors Gynecol Oncol 2005 97 310 7 10.1016/j.ygyno.2005.01.010 15863123

[B16] Ferrandina G Mantegna G Petrillo M Fuoco G Venditti L Terzano S Moruzzi C Lorusso D Marcellusi A Scambia G Quality of life and emotional distress in early stage and locally advanced cervical cancer patients: a prospective, longitudinal study Gynecol Oncol 2012 124 389 394 10.1016/j.ygyno.2011.09.041 22035809

[B17] Ferrandina G Legge F Fagotti A Fanfani F Distefano M Morganti A Cellini N Scambia G Preoperative concomitant chemoradiotherapy in locally advanced cervical cancer: safety, outcome and prognostic measures Gynecol Oncol 2007 107 S127 S132 10.1016/j.ygyno.2007.07.006 17727936

[B18] Legge F Margariti PA Lucidi A Macchia G Petrillo M Iannone V Carone V Morganti AG Scambia G Ferrandina G Completion surgery after concomitant chemoradiation in obese women with locally advanced cervical cancer: Evaluation of toxicity and outcome measures Acta Oncol 2013 52 166 173 10.3109/0284186X.2012.698753 22746313

[B19] Ferrandina G Lucidi A Paglia A Corrado G Macchia G Tagliaferri L Fanfani F Morganti AG Valentini V Scambia G Role of comorbidities in locally advanced cervical cancer patients administered preoperative chemoradiation: impact on outcome and treatment-related complications Eur J Surg Oncol 2012 38 238 244 10.1016/j.ejso.2011.12.001 22200246

[B20] Zigmond AS Snaith RP The Hospital Anxiety and Depression scale Acta Psychiatr Scand 1983 67 361 370 10.1111/j.1600-0447.1983.tb09716.x 6880820

[B21] Costantini M Musso M Viterbori P Bonci F Del Mastro L Garrone O Venturini M Morasso G Detecting psychological distress in cancer patients: validity of the Italian version of the Hospital Anxiety and Depression Scale Support Care Cancer 1999 7 121 127 10.1007/s005200050241 10335929

[B22] Aaronson NK Ahmedzai S Bergman B Bullinger M Cull A Duez NJ Filiberti A Flechtner H Fleishman SB de Haes JC The European Organization for Research and Treatment of Cancer QLQ-C30: a quality-of-life instrument for use in international clinical trials in oncology J Natl Cancer Inst 1993 85 365 376 10.1093/jnci/85.5.365 8433390

[B23] Greimel ER Kuljanic Vlasic K Waldenstrom AC Duric VM Jensen PT Singer S Chie W Nordin A Bjelic Radisic V Wydra D European Organization for Research and Treatment of Cancer Quality-of-Life Group **The European Organization for Research and Treatment of Cancer (EORTC) Quality-of-Life questionnaire cervical cancer module: EORTC QLQ-CX24** Cancer 2006 107 1812 1822 10.1002/cncr.22217 16977652

[B24] Cocks K King MT Velikova G Martyn St-James M Fayers PM Brown JM Evidence-based guidelines for determination of sample size and interpretation of the European Organisation for the Research and Treatment of Cancer Quality of Life Questionnaire Core J Clin Oncol 2011 29 89 96 10.1200/JCO.2010.28.0107 21098316

[B25] Osoba D Rodrigues G Myles J Zee B Pater J Interpreting the significance of changes in health-related quality-of-life scores J Clin Oncol 1998 16 139 144 9440735 10.1200/JCO.1998.16.1.139

[B26] Ringash J O'Sullivan B Bezjak A Redelmeier DA Interpreting clinically significant changes in patient-reported outcomes Cancer 2007 110 196 202 10.1002/cncr.22799 17546575

[B27] Lutgendorf SK Anderson B Ullrich P Johnsen EL Buller RE Sood AK Sorosky JI Ritchie J Quality of life and mood in women with gynecologic cancer: a one year prospective study Cancer 2002 94 131 140 10.1002/cncr.10155 11815969

[B28] Petersen RW Quinlivan JA Preventing anxiety and depression in gynaecological cancer: a randomised controlled trial BJOG 2002 109 386 394 10.1111/j.1471-0528.2002.01271.x 12013159

[B29] Suzuki N Ninomiya M Maruta S Hosonuma S Nishigaya Y Kobayashi Y Kiguchi K Ishizuka B Psychological characteristics of Japanese gynecologic cancer patients after learning the diagnosis according to the hospital anxiety and depression scale J Obstet Gynaecol Res 2011 37 800 808 10.1111/j.1447-0756.2010.01437.x 21450027

[B30] Andersen BL Farrar WB Golden-Kreutz D Emery CF Glaser R Crespin T Carson WE 3rd Distress reduction from a psychological intervention contributes to improved health for cancer patients Brain Behav Immun 2007 21 953 961 10.1016/j.bbi.2007.03.005 17467230 PMC2039896

[B31] Dursun P Ayhan A Kuscu E Nerve-sparing radical hysterectomy for cervical carcinoma Crit Rev Oncol Hematol 2009 70 195 205 10.1016/j.critrevonc.2008.09.003 18926716

